# Types and characteristics of shared microbiota within families of ethnic minorities in Inner Mongolia

**DOI:** 10.1128/spectrum.00706-25

**Published:** 2026-01-28

**Authors:** Caiqing Yao, Lijun You, Jing Li, Jingjing E, Junguo Wang

**Affiliations:** 1 Key Laboratory of Dairy Biotechnology and Engineering, Ministry of Education, Inner Mongolia Agricultural University 117454 , Hohhot, China; 2 College of Food Science, Shanxi Normal University 47842, Taiyuan, China; 3 School of Food Science and Engineering, Bohai University 12687, Liaoning, China; Yangzhou University, Yangzhou, Jiangsu, China

**Keywords:** gut microbiota, gut microbial diversity, family, shared microbiota

## Abstract

Gut microbial diversity is often studied from the perspective of community composition, but less is known about the genetic heterogeneity within species. The human gut microbiota exhibits substantial individual variation, influenced by numerous factors. This study characterized the gut microbial composition of 25 ethnic minority families from Inner Mongolia. We found that cohabitation within the same family was a more significant determinant of variation in both macrodiversity and microdiversity than other factors, including family role, ethnicity, sex, age, and geographical region. Based on average nucleotide identity, we identified 17 bacterial strains that were shared within families. Among these, *Bifidobacterium adolescentis* and *Holdemanella biformis* demonstrated higher average abundance, while *Eubacterium rectale* and *Clostridiales bacterium* were shared more frequently and across multiple families. Annotation against the Kyoto Encyclopedia of Genes and Genomes (KEGG) database revealed that most of these shared strains possess the genetic potential for the production or biosynthesis of short-chain fatty acids (SCFAs). Concurrently, computational analysis demonstrated that the genes of shared strains showed relatively low non-synonymous to synonymous polymorphism (pN/pS) ratios. Correlation network analysis indicated that, with the exception of *Clostridiales bacterium*, the shared strains generally did not occupy central nodes within the microbial interaction network. This research provides valuable insights into the patterns of gut microbiota sharing within families of Inner Mongolian ethnic minorities and offers a scientific basis for developing family-scale probiotic interventions.

**IMPORTANCE:**

China is a multi-ethnic nation with the Han ethnic group as the majority. Among its diverse ethnic groups, the populations of the Daur, Evenki, and Buryat are relatively small, with the Seventh National Population Census reporting their numbers at 132,299, 34,617, and 436,000, respectively. These minority groups often consume a diet characterized by high fat, high protein, and low fiber, primarily consisting of dairy products and meat, which may elevate their risks of obesity, diabetes, and cardiovascular diseases. Investigating the shared gut microbiota of these local ethnic minorities at both macro and micro diversity levels, using the family as a unit, is instrumental in safeguarding their health. In the future, based on this study, guidelines can be formulated to either facilitate or hinder the sharing of microbiota, depending on whether a specific shared strain is identified as beneficial or risky to health.

## INTRODUCTION

Gut microbes have important roles in health and disease, and the microbiota contributes to human metabolism and the development of the nervous and immune systems, thereby influencing physical and mental health (1–5). China is a multi-ethnic country. In Hulunbuir, Inner Mongolia Autonomous Region, there are three relatively unique ethnic groups living there, namely, the Daur ethnic group, the Ewenki ethnic group, and the Buryat ethnic group. According to the Seventh National Population Census, the population of these ethnic groups is relatively small. Historically, they have lived in close proximity and intermingled with one another, maintaining frequent interactions. There are also similarities in lifestyle, literature, art, customs, and other aspects. Moreover, the average temperature of the region is low throughout the year, and the degree of industrialization is low. The local people primarily engage in animal husbandry, and their diet consists primarily of dairy and meat. The local ethnic minorities have developed unique gut microbiota due to their distinct lifestyles, living environments, and dietary habits. Since the population size of this research group was small, and there were difficulties involved in sampling, the gut microbial composition of these unique populations has not been reported yet. In-depth exploration of the characteristics of the gut microbiota of ethnic minorities is of particular significance for protecting the health of ethnic minority groups.

Many host factors, ranging from racial and geographical disparities to cultural and behavioral characteristics, affect the diversity and composition of the human gut microbiota on a global scale today (6). Among them, geographical, ecological, and population-specific factors, especially host genetics and race, contribute to explaining the variations in the diversity and composition of the human gut microbiota. However, covariates associated with race or geographical location, such as diet, culture, and behavioral habits, may confound a clear understanding of the key drivers of the significant geographical differences reported in human population studies (7, 8). It has been reported that the microbial communities found in the guts of genetically related people tend to be more similar than those of unrelated people (9). However, explorative analyses of microbial genotype distribution and transmission give credit to cohabitation rather than a kinship, for this similarity. Cohabitation is a crucial covariate for strain transmission and a driving factor for the sharing of individual microbiome characteristics (10, 11). This indicates that shared environmental factors or lifestyle (e.g., exposure to the same microbial sources, diet, or circadian rhythms) are more influential in shaping gut microbiota than genetics. In another unpublished study, we conducted a comprehensive analysis of the characteristics of the gut microbiota of the local ethnic minority population. This study focuses on analyzing the shared microbiota within local ethnic minority families and its characteristics. The objective is to provide a theoretical basis for targeted probiotic interventions at the family level and contribute to protecting the gut health of ethnic minority groups.

In recent times, the research on gut microbes based on the family unit generally adopts 16S rRNA amplicon sequencing, differences in the relative abundance of amplicon sequence variants (ASVs; Bray-Curtis dissimilarity), and differences in the presence/absence of ASVs (Jaccard dissimilarity) were previously thought to represent the proportion of unshared taxa between two individuals (12); however, this view is clearly inaccurate. The species concept was translated into the bacterial context as a group forming a coherent genomic cluster. Despite this genetic similarity, it was also established that a large magnitude of phenotypic variance is possible among strains from the same species (conspecific strains) (13). The relationship between strain identity and host health demonstrates the fact that it is not sufficient to study microbial communities at species-level resolution. Furthermore, studying within-species variation has traditionally been limited to culturable bacterial isolates, low-resolution microbial community fingerprinting, and metagenomic sequencing. However, technological advances have enabled culture-free, high-resolution strain and subspecies analysis in high-throughput and complex environments (13), enabling the study of macrodiversity (inter-species diversity) and microdiversity (intra-species genetic diversity).

This study investigated the gut microbiota of 25 family members of ethnic minorities in Inner Mongolia. Based on the metagenomic deep sequencing technology, the research was conducted focusing on two perspectives: the macrodiversity and microdiversity of the gut microbiota. The gut microbial composition of an understudied population has been reported in this study. Furthermore, the strains likely to be shared among family members have been identified, and their abundance, prevalence, functional potential, and selective pressure have been investigated.

## MATERIALS AND METHODS

### Subject recruitment

**Fig 1:**
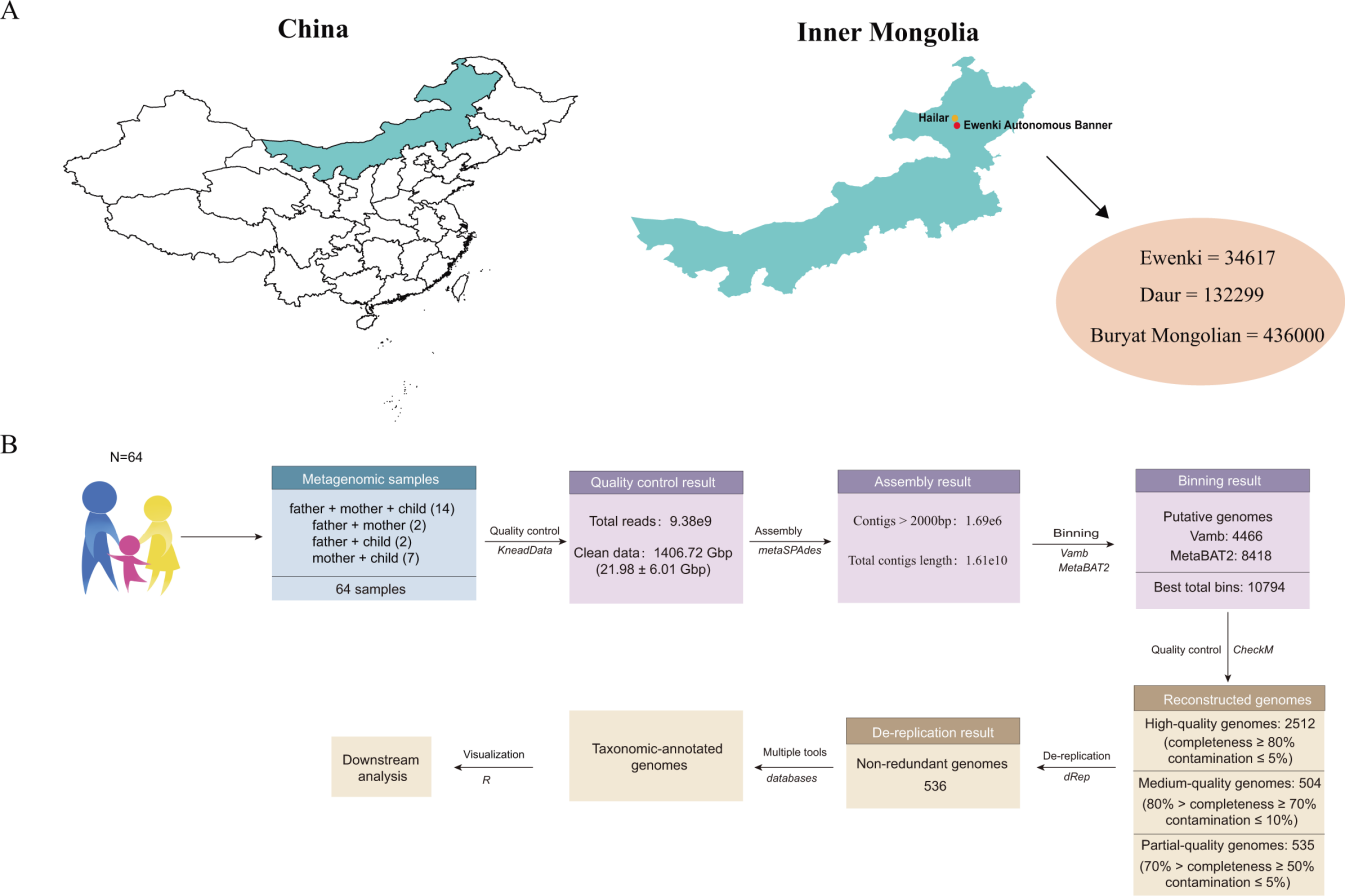
(**A**) Geographic distribution of the 64 volunteers. The map was generated using the maps package (version 3.3.0) in R. (**B**) Data analysis process.

Between 1 August and 30 August 2019, 73 healthy minority volunteers whose families had resided in Hulunbuir for over three generations were recruited. Due to some participants dropping out during the study, 64 subjects who met the criterion of having at least two enrolled members from the same family were ultimately selected for the trial (Fig. 1A). Please see the attached table for detailed information on the population (Table S1). All participants signed the informed consent form before the study started. All subjects had no gastrointestinal symptoms and had not used antibiotics or probiotics within the 6 months preceding the study. The subjects were required to self-collect fecal samples into airtight sterile stool specimen collection tubes provided in advance and return them to the research team. Samples were then frozen immediately in liquid nitrogen and stored at −80°C until further analysis.

### Metagenomic DNA extraction and sequencing

Stool samples were thawed on ice for 1 h, and the DNA was extracted using a standard QIAamp Fast DNA Stool Mini Kit (Qiagen, Hilden, Germany) following the manufacturer’s instructions. The quality and concentration of DNA were detected by gel electrophoresis and Nanodrop spectrophotometer (Thermo Electron Corp., Waltham, MA, USA), respectively. It was ensured that the DNA concentration was above 100 ng/μL and the 260 nm/280 nm ratio was between 1.8 and 2.0. All the samples were sequenced using the Illumina HiSeq 2500. After quality control and human DNA removal, a total of 1406.72 Gbp data volume and 9.38 × 10^9^ high-quality end reads were obtained. An average of 21.98 ± 6.01 Gbp data was obtained from each sample (Fig. 1B).

### Metagenomic assembly, contig binning, genome dereplication

The reads from each sample after quality control were assembled using MEGAHIT (14). For the assembled contigs, binning was performed for those with lengths >2 000 bp. Metagenome-assembled genomes (MAGs) were obtained through VAMB (15) and MetaBAT2 (16). Finally, 10,794 MAGs were screened out through MetaWRAP (17). Then, sample reads were mapped to contigs by BWA-MEM (18), and the read depth of each sample was calculated by using the jgi_summarize_bam_contig_depths function in MetaBAT2, which used Samtools (19). CheckM (20) was used to evaluate the completeness and contamination of MAGs, and following the standard of Parks et al. (21), 2,512 high-quality MAGs, 504 medium-quality MAGs, and 535 low-quality MAGs were obtained. Then, 536 species-level genomic bins (SGBs) were generated from 2,512 MAGs of high quality by clustering with dRep (-pa 0.95 -sa 0.95) (22) (Fig. 1B; Table S2).

### Taxonomic annotation, abundance, pi, and pN/pS of species-level genomic bins

The SGBs were annotated through Kraken2 (23) and NCBI Nonredundant Nucleotide Sequence Database. Putative genes in Contigs were predicted by Prodigal (24), and the predicted genes were searched against the UniProt Knowledgebase. Each SGB abundance was calculated by CoverM (https://github.com/wwood/CoverM) and expressed in reads per kilobase million (RPKM). InStrain (25) was used to calculate the nucleotide diversity and non-synonymous to synonymous polymorphism (pN/pS) of the SGBs.

### Genome filtering and comparison

A total of 3,787 MAGs with completeness >=50% and contamination rate <=10% were selected from 10,794 raw MAGs for inter-genome comparison using the compare function of dRep (22), and the parameter was set as' -pa 0.9 -sa 0.95 --S_algorithm fastANI '. According to the method of Crits-Christoph et al. (26), the MAGs (1,312 in total, Table S3) assembled in at least two or more members in 30% of the 25 families were calculated whether they were shared MAGs within families, and the prevalence rate and sharing times among families were calculated.

### Content and prediction of metabolic modules of shared species populations within the family

For the MAGs shared within families, each representative species population identified through annotation was named an ANG, and CoverM was used to calculate the abundance of each ANG. Predictions of key metabolic modules were obtained by comparing Prodigal’s gene prediction results with the Kyoto Encyclopedia of Genes and Genomes (KEGG) Orthologies (KOs) database. This study focused on the production and synthesis of short-chain fatty acids (27). All pathways were detected by Omixer-RPM (28) and defined in ANGs (parameter: -c 0.66).

### Gene function annotation of shared MAGs within family

The genes of carbohydrate-active enzymes (CAZymes) carried by MAGs were annotated using the dbCAN2 database with HMMER (29). Functional gene annotation was performed using the Kyoto Encyclopedia of Genes and Genomes (KEGG) database with Usearch (30). Resistance gene annotation was carried out by comparing with the CARD database using Blastp (31).

### Statistical analyses

All statistical analyses were performed using the R software (v.4.0.3). The Shannon index was performed with R package vegan (v.2.5-1). Bray-Curtis distance within and between families was performed with R package vegdist. PERMANOVA with marginal effects tests (999 permutations) was used to evaluate differences in gut microbiota communities of samples at family, family role, ethnicity, sex, age, and region. Spearman’s correlation analysis was used to identify the correlations among strains. All graphical presentations were generated in the R and Adobe Illustrator (AI) environments.

## RESULTS

### Composition of gut microbes at the strain level in ethnic minorities

**Fig 2:**
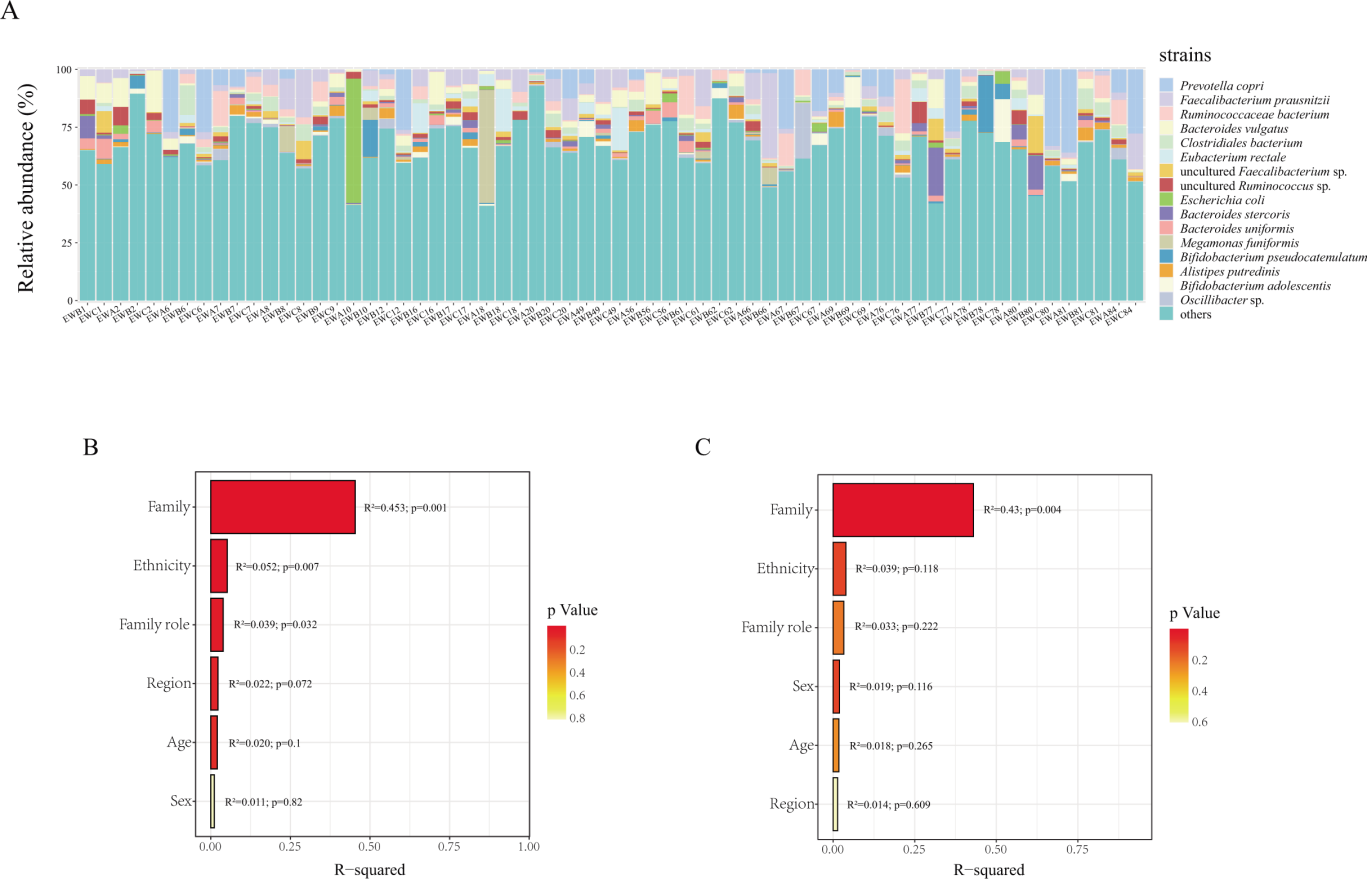
(**A**) Microbial composition at the strain level of the 64 samples. (**B**) The independent contribution of each factor to microbial community variation was assessed using PERMANOVA with marginal effects tests (999 permutations), controlling for all other variables. Bars indicate the proportion of variance explained (*R*²), and *P*-values denote the significance of each factor’s marginal effect. (**C**) The independent contribution of each factor to microbial intraspecies genetic variation was assessed using PERMANOVA with marginal effects tests (999 permutations), controlling for all other variables. Bars indicate the proportion of variance explained (*R*²), and *P*-values denote the significance of each factor’s marginal effect.

Here, we first aim to gain a comprehensive understanding of the gut microbiota in ethnic minority populations. The abundance of each type of microorganism was obtained by aggregating the identified strains. The dominant strains present in the gut of minority populations included *Prevotella copri* (5.34%), *Faecalibacterium prausnitzii* (4.88%), *Ruminococcaceae bacterium* (3.07%), *Bacteroides vulgatus* (3.05%), *Clostridiales bacterium* (2.23%), *Eubacterium rectale* (1.87%), uncultured *Faecalibacterium* sp. (1.46%), uncultured *Ruminococcus* sp. (1.44%), *Escherichia coli* (1.36%), *Bacteroides stercoris* (1.36%), *Bacteroides uniformis* (1.28%), *Megamonas funiformis* (1.26%), *Bifidobacterium pseudocatenulatum* (1.23%), *Alistipes putredinis* (1.22%)*, Bifidobacterium adolescentis* (1.21%), and *Oscillibacter* sp. (1.06%) with an average relative abundance of more than 1% (Fig. 2A).

### Major factors affecting the composition of gut microbes in ethnic minorities

The PERMANOVA with marginal effects tests (999 permutations) was performed on the differences in microbial community composition and genetic heterogeneity within species of samples from different families, family role, ethnicity, sex, age, and region. For microbial community composition, the family factor demonstrated a significantly higher explanatory power (*R*² = 0.453; *P* = 0.001) than any other factors, identifying that it was the most important factor causing differences in the composition of gut microbes in the population (Fig. 2B). A consistent result was observed for genetic heterogeneity within species (*R*² = 0.43; *P* = 0.004), also confirming that the family is the dominant factor shaping intra-species genetic heterogeneity in the human gut microbiota (Fig. 2C).

### Similarity of gut microbes in ethnic minority populations

**Fig 3:**
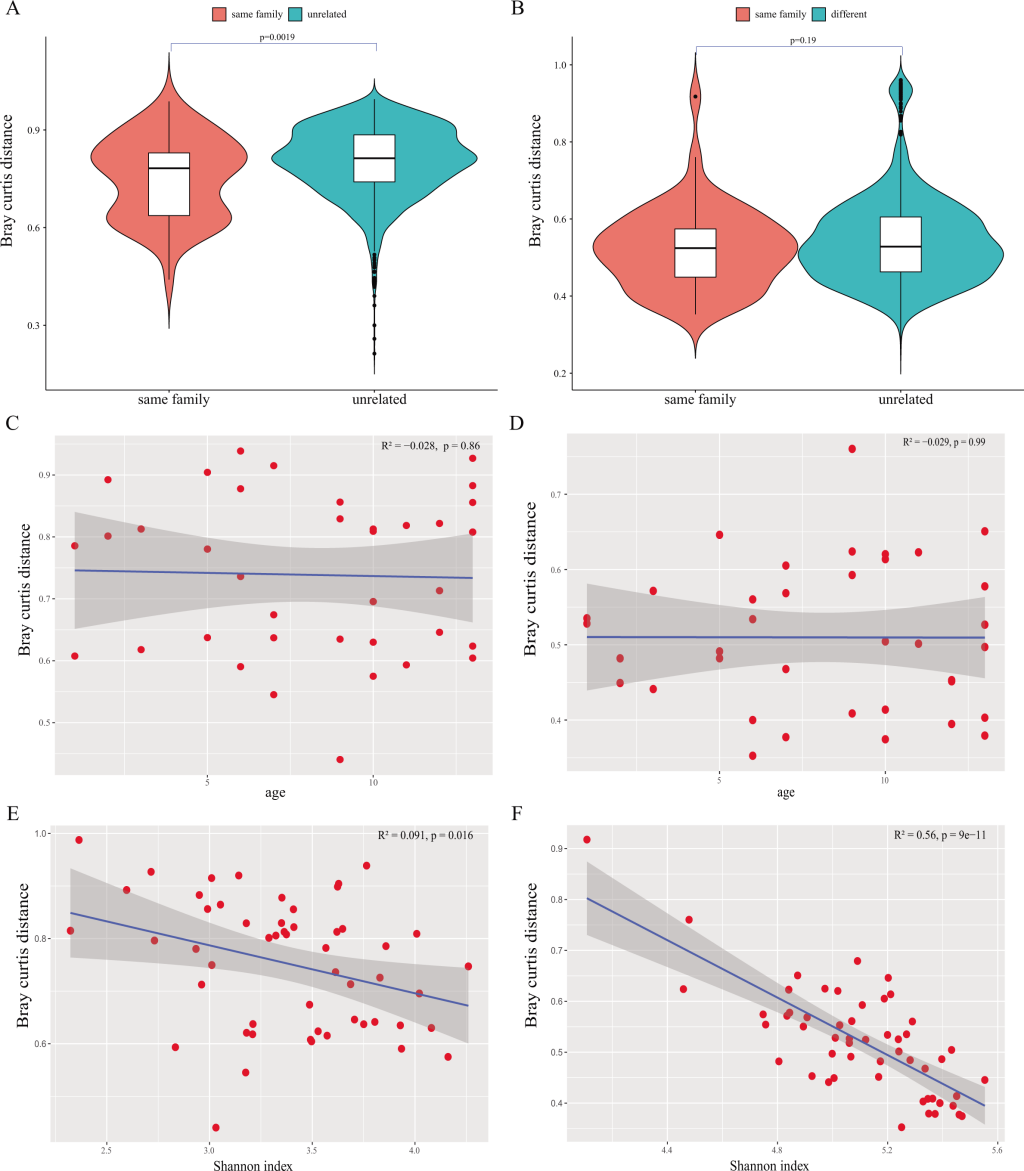
(**A**) Bray-Curtis dissimilarity of gut microbiota within and between different families, at the level of microbial community composition. (**B**) Bray-Curtis dissimilarity of gut microbiota within and between different families, at the level of intraspecies genetic composition. (**C**) Correlation between children’s age and the Bray-Curtis distance between children and their parents in terms of microbial composition, *P*-values from the Wilcoxon test, and the Spearman’s correlation coefficient (*R*^2^) are shown in the figure. (**D**) Correlation between children’s age and Bray-Curtis distance between children and their parents’ gut microbes in terms of microbial intraspecies genetic composition, *P*-values from the Wilcoxon test, and the Spearman’s correlation coefficient (*R*^2^) are shown in the figure. (**E**) The correlation between the microbial diversity of family members and the Bray-Curtis distance of gut microbes between paired members within the family. The *X*-axis refers to the average value of the Shannon index of paired members within the family, and the *Y*-axis refers to the Bray-Curtis distance of gut microbes between paired members within the family. (**F**) The correlation between the microbial diversity of family members and the Bray-Curtis distance of gut microbial genetic composition between paired members within the family. The *X*-axis refers to the average value of the Shannon index of paired members within the family, and the *Y*-axis refers to the Bray-Curtis distance of gut microbial genetic composition between paired members within the family.

Considering that family factors were the most crucial factor causing the differences in gut microbiota between and within species, the similarity of microbiota within and between families was calculated. The calculations revealed that the difference in microbial species composition among members of different families was greater than that among members of the same family (*P* < 0.01), indicating that the gut microbial composition among family members was more similar than that among unrelated people (Fig. 3A). However, the level of genetic heterogeneity among within-family members was comparable to that among members from different families (Fig. 3B). The study also found that as the children grew older, the similarity to their parents’ microbial composition remained unchanged, both at the interspecies and intraspecies levels (Fig. 3C and D). Interestingly, in families with high α diversity, there was a higher degree of similarity in gut microbial composition and gene composition among family members (Fig. 3E and F), indicating that highly diverse microbial communities may be more easily shared within families.

### Microbiomes shared within families

**Fig 4:**
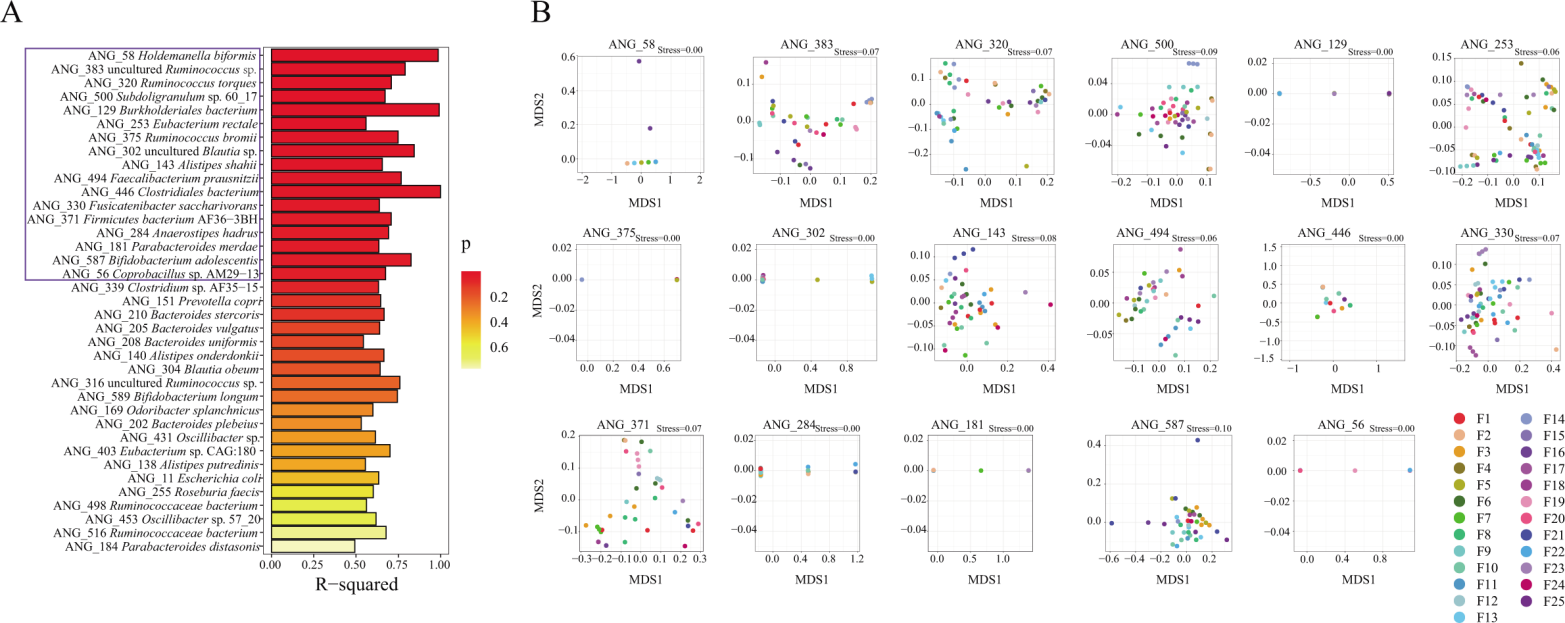
(**A**) Percentage of variation in shared genome genetic similarity (ANI) explained by the family factor. (**B**) Multidimensional scaling of genetic differences between genomes shared within families. The first two axes are plotted. There is a single point for each genome independently assembled for a population, and the genomes are colored by family origin.

In this study, genomes that were present in at least two members within a family and were present in at least 30% of all families were calculated to observe whether family factors predicted the genetic similarity of the assembled genomes. We tested if genomes obtained from the same family were more similar than those from different families. We found that the genetic variation of genomes from 17 of the 39 populations was significantly associated with family (*R*^2^ *>* 0.5; *P <* 0.05) (Fig. 4A; Table S4). These genomes were identified as *Holdemanella biformis*, uncultured *Ruminococcus* sp., *Ruminococcus torques*, *Subdoligranulum* sp. 60_17, *Burkholderiales bacterium*, *Eubacterium rectale*, *Ruminococcus bromii*, uncultured *Blautia* sp., *Alistipes shahii*, *Faecalibacterium prausnitzii*, *Clostridiales bacterium*, *Fusicatenibacter saccharivorans*, *Firmicutes bacterium* AF36-3BH, *Anaerostipes hadrus*, *Parabacteroides merdae*, *Bifidobacterium adolescentis*, *Coprobacillus* sp. AM29-13 (Table S5). Among them, *Clostridiales bacterium*, *Burkholderiales bacterium,* and *Holdemanella biformis* explained more than 98% of the variance (Fig. 4A). The multidimensional scaling (MDS) plot derived from the genomic nucleotide identity matrix for each population showed a clear correlation with the family (Fig. 4B). Thus, the influence of family factors in the genetic variation of certain individual species is demonstrated.

### Abundance, prevalence, and functional potential of microbial communities shared within families

**Fig 5:**
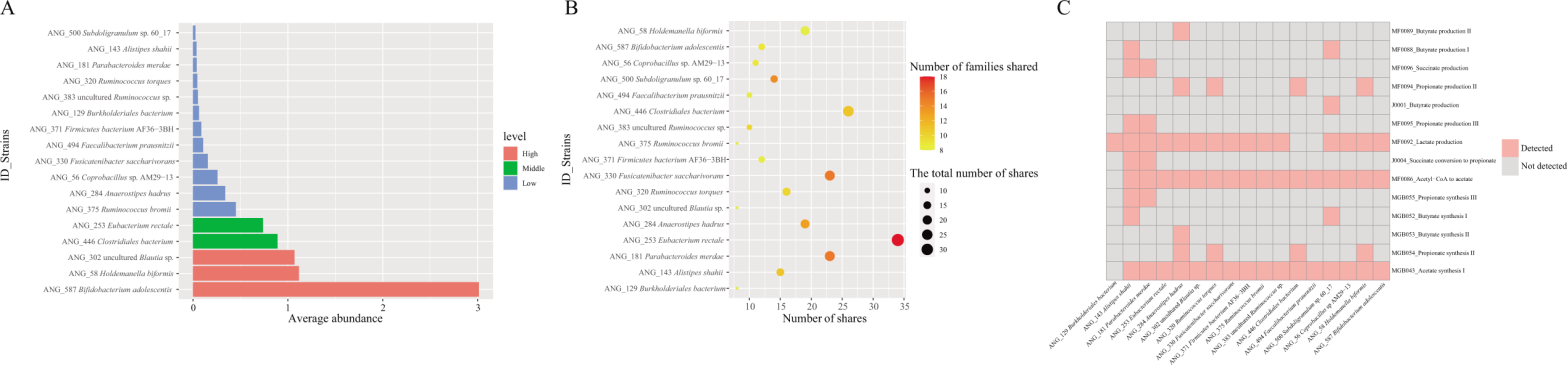
(**A**) The average content of the shared microbiota genome within a family in the sample. (**B**) The number of family prevalence of the bacterial genome shared within the family and the number of times it was shared in the family. (**C**) Heatmap showing the presence and absence of key metabolic modules. Pink cells indicate the module was detected in the sample, while gray cells indicate it was not detected.

The study found that *Bifidobacterium adolescentis*, *Holdemanella biformis*, and uncultured *Blautia* sp. had the highest relative abundance among the 17 easily shared strains (Fig. 5A). Furthermore, *Eubacterium rectale*, *Clostridiales bacterium*, *Fusicatenibacter saccharivorans,* and *Parabacteroides merdae* were shared more than 20 times within families and were prevalent in multiple families (Fig. 5B). The highest abundance of *Bifidobacterium adolescentis* was shared 12 times and occurred in 9 different families. Among the strains that were easy to share, except for *Burkholderiales bacterium*, it was predicted by the KEGG database that the remainder possess the potential for SCFAs production or biosynthesis (Fig. 5C).

### Selective pressure on the function of microbiome genes shared within families

**Fig 6:**
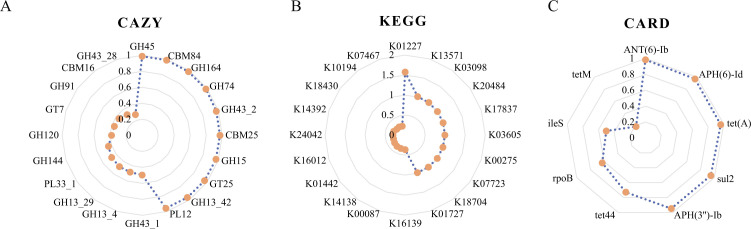
(**A**) Carbohydrate active enzymes (CAZymes) annotation results corresponding to the top 10 and bottom 10 genes with the non-synonymous to synonymous polymorphism (pN/pS) value of the microbial community genes shared within the family. (**B**) Functional annotation results corresponding to the top 10 and bottom 10 genes with the pN/pS value of the microbial community genes shared within the family. (**C**) The drug resistance gene annotation results corresponding to the pN/pS value of the microbial community genes shared within the family.

The selective pressure acting upon genes in the genomes of 17 microbial communities (Table S6) was examined and only functions annotated by genes with the most extreme (highest and lowest) pN/pS values are shown in the figure. It was found that all CAZymes genes and drug resistance genes were subjected to purifying selection (pN/pS<1) (Fig. 6A and C). Furthermore, annotated by the KEGG database, it was found that three pathways, K01227, K13571, and K03098, were under positive selective pressure (pN/pS>1) (Fig. 6B).

### Correlation among gut microbial communities in ethnic minority populations

**Fig 7:**
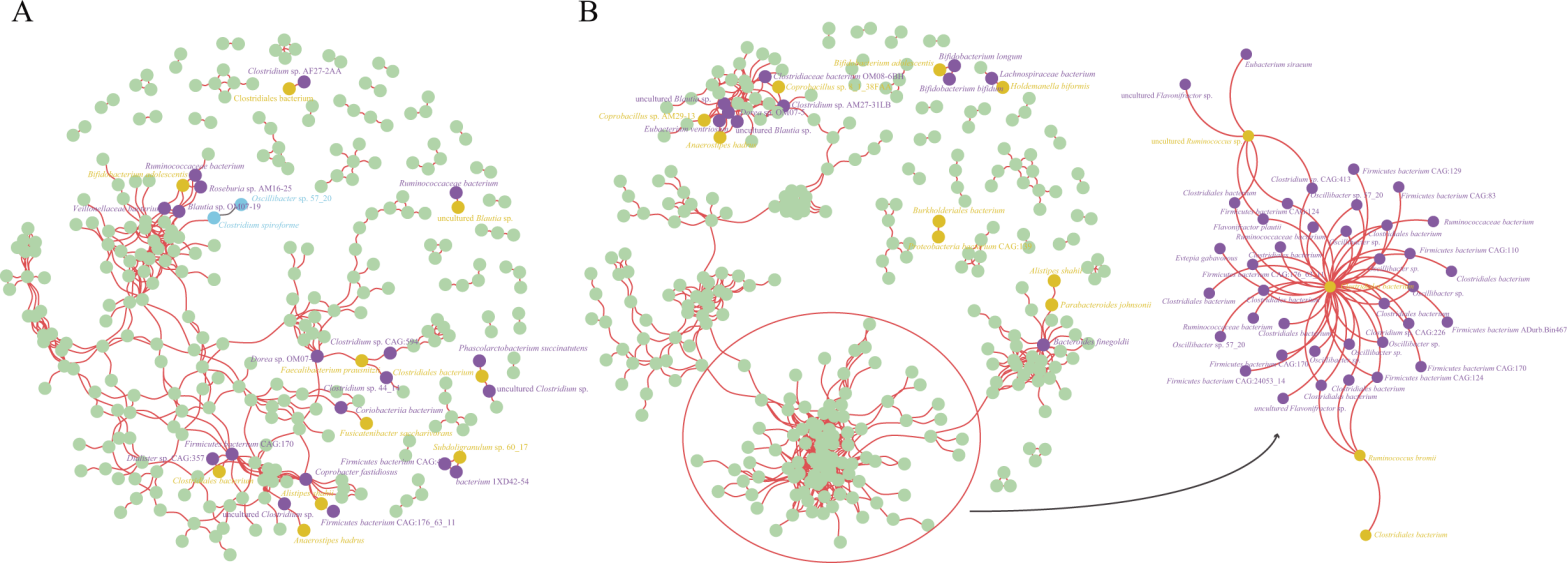
(**A**) Correlation among strains at the level of microbial composition, yellow: microbiome shared within the family, purple: strains with positive correlation with the microbiome shared within the family, blue: microbes with negative correlation. (**B**) Correlation between strains at the level of microbial intraspecies genetic composition, yellow: microbiome shared within a family, purple: strains positively correlated with a microbiome shared within a family. (The figure shows strains with a correlation coefficient of 0.6 rspearman|< 1)

The interspecies and intraspecies diversity of microorganisms was used to calculate the correlation between the bacterial communities. The graph shows the correlations between the 17 microbiota shared within the family and other microbiota. In terms of microbiota composition, the correlation between almost all bacteria was positive (0.6 < rspearman < 1, *P* < 0.05). The core nodes were defined as the points having the most correlation relationships with the remaining microorganisms. The study found that none of the strains shared within the family were at the core node of the relationship (Fig. 7A). In terms of genetic diversity of the microbiota, only the *Clostridiales bacterium* was at the core node of the relationship. There were 30 strains positively correlated with *Clostridiales bacterium*, among which *Oscillibacter* sp., *Clostridiales bacterium,* and *Firmicutes bacterium* had more strains (Fig. 7B).

## DISCUSSION

Typically, the family factor is the most important in shaping the interspecies and intraspecies diversity of gut microbes, among various factors, including family role, ethnicity, sex, age, and region. The families recruited in the study not only have the same genetic background, but their living environment, habits, and dietary conditions are more similar than other unrelated families. Studies in human and domestic animal models indicate that related individuals and those who spend significant time in physical contact generally have more similar gut microbiomes (32). Yatsunenko et al. reported that the gut microbiota of adolescents and their parents were more similar than that of unrelated adults, and the gut microbiota between parents was also more similar, consistent with the findings of this study (33). Notably, the genetic heterogeneity within the microbial species among members of the same family was not found to be more similar than that among members of different families. However, the explanatory power of the family factor among many factors is still far greater than other factors. Strain-level microbial genomic variation typically consists of single-nucleotide variants (SNVs) as well as acquisition/loss of genomic elements including genes, operons, or plasmids, which is complex (34). Ellegaard et al. illustrated that the gut microbiota of closely related animal hosts can differ vastly in genomic diversity while displaying similar levels of diversity based on the 16S rRNA gene (35). This is also a reminder of the fact that genetic heterogeneity within the strain cannot be ignored. In addition, the children in this study were all under 13, and no particular trend was found between the children’s age and the similarity with their parents’ gut microbiota. Korpela et al. also reported that the influence of family environment peaked between the ages of 2 and 10. After 10, the strain sharing was generally lower but still significantly higher than that of unrelated individuals (36). Additionally, the study also found that the higher the diversity of individual microbes, the greater was the similarity of the gut microbes among family members. In a study of gregarious wild baboons, sociability was associated with an increase in gut microbial α diversity, suggesting that transmission contributed to an increase in gut microbial diversity. Conversely, an increase in gut microbial α diversity was also closely related to an increase in bacterial transmission (37).

The dominant strains in the gut of local ethnic minority populations are *Prevotella copri* and *Faecalibacterium prausnitzii*. These two strains were reported to be the dominant species in the gut microbiota of healthy Asian adults (38). Of these, *Faecalibacterium prausnitzii* could be easily shared within the family. Among these easily shared species, all except *Burkholderiales bacterium* were predicted to be potentially beneficial microbiota capable of producing or synthesizing SCFAs in the gut. Although it could not be guaranteed that all the identified easy-to-share strains were due to the transmission among members, the transmission of strains between members must exist. On the one hand, bacteria transmitted through social interactions can enhance the host’s resistance to pathogens and stimulate the host’s immunity (39, 40). Meanwhile, they can also outcompete pathogens in resource competition or produce by-products that completely inhibit pathogens. These gut bacteria transmitted through the social environment are beneficial to personal health (41, 42). In addition, over time, frequent social transmission may increase microbial diversity and, to some extent, improve the host’s health status (43). *Burkholderiales bacterium* has been shown to be enriched in the gut microbiota of patients with inflammatory bowel disease (44); these bacteria have multidrug resistance, transmissibility, and biofilm formation ability (45). Studies have shown that the bacteria can reduce respiratory function in patients and has spread to all regions of the Russian Federation except the Far East (46); in the future, the spread of this bacterium among the population should be controlled. The *Clostridiales bacterium*, which is extensively shared within and prevalent in multiple families, was identified as the most active microbial component of the gut environment in healthy adults. *Clostridium-*induced endospore formation can facilitate its horizontal transfer in the environment (36). The evaluation of the correlation between bacterial species revealed that *Clostridiales bacterium* was not only at the core node of the network relationship but also promoted the mutual growth of various bacteria under the Firmicutes phylum. Hildebrand et al. found that Firmicutes, which are rich in spore-forming genes, also have a strong transmission strategy for family transmission (47).

The genes of easily shared microorganisms in the gut of minority populations have relatively low pN/pS ratios and are constant across hosts, which indicates the presence of similar selective limitations between individuals. Thus, the evolution of gut species might be dominated by long-term purging selection and drift instead of rapid adaptation to specific host environments (48). This result might be related to the ideological tradition of the local ethnic minorities, the absence of immigrant populations, and permanent residence. Additionally, larger pN/pS ratios might be related to the tendency for codon mutations, while promoter and terminator mutations could be associated with potential functional defects in the gut microbiota, leading to multiple chronic diseases (49). Since the samples in this study were all from the gut of healthy people, their pN/pS ratios were low.

The expression of millions of genes and the differences in the functions of countless genes in the gut microbiome are one of the sources of human genetic and metabolic diversity. Ethnic minority populations often adhering to high-fat, high-protein, and low-fiber diets (primarily consisting of dairy and meat products) may face increased risks of obesity, diabetes, and cardiovascular diseases. Probiotic interventions can help prevent these conditions, and the family unit serves as a key factor influencing the structure and stability of gut microbiota. Implementing probiotic interventions at the household level can facilitate the transmission and colonization of bacterial strains among members, thereby enhancing intervention efficacy and establishing a beneficial cycle of “family microecology.” However, unlike skin microbes, the true niche of gut-shared microbes in humans is not after direct contact. Therefore, it is difficult to ascertain whether the microbes shared within the family in this study came from direct and frequent contact with cohabitants. Detecting familial microbiome community patterns does not necessarily reflect the actual transmission of microorganisms. It could also result from the shared genetic backgrounds and cultural transmission of lifestyle and dietary habits that are selective for a similar microbial composition (50, 51). Furthermore, a key limitation of this study lies in the resolution of MAGs. For a subset of MAGs, precise taxonomic assignment at the species or strain level was not achievable, which hindered a more detailed interpretation of the sharing analysis. For these broadly defined taxonomic groups, we cannot rule out the possibility that family members harbored distinct strains within the same clade, which may possess divergent functional characteristics. Therefore, the “sharing” observed at this level should be considered a conservative estimate of true strain-level transmission. Future research integrating long-read sequencing or cultivation of these difficult-to-classify microbial taxa will be crucial for refining our understanding of within-family microbial sharing at the ultimate resolution. Additionally, it should be noted that the functional insights regarding SCFAs production were derived from *in silico* predictions based on the KEGG database. While this approach effectively identifies the genetic potential of shared strains, it does not directly confirm that these shared microbes actively produce SCFAs. To explicitly validate these predictions, future work should prioritize the pure culture isolation of these key bacterial species, followed by experimental verification of their SCFAs production capability.

### Conclusion

In the study of the macrodiversity and microdiversity of the gut microbiota of ethnic minorities at the family level, it was found that the family environment is an important factor among the many factors influencing the heterogeneity of the gut microbiota of ethnic minorities. The composition of gut microbes in the same family is more similar than that between different families. Additionally, the study’s findings revealed 17 species of microbiota that are easily shared within the family, most of which were predicted—based on database annotations—to be potential probiotics with the ability to produce or synthesize short-chain fatty acids, and their genes are subject to purifying selection. The findings reported in this study provide a theoretical reference for future targeted probiotic interventions for local populations based on family units.

## Data Availability

The raw metagenomic sequencing reads generated in this study have been deposited in the NCBI SRA database under BioProject accession PRJNA919082. The complete set of assembled MAGs (in FASTA format) is publicly available in the Zenodo repository (https://zenodo.org/records/18105277). All associated metadata, software and database version details, and custom analysis scripts are publicly available in the GitHub repository (https://github.com/1140054471/family-shared-microbiota-mag-pipeline) and have been archived with a permanent DOI on Zenodo (https://zenodo.org/records/18067692).

## SUPPLEMENTAL MATERIAL

Supplemental tables spectrum.00706-25-s0001.xlsx Tables S1 to S6.

ASM does not own the copyrights to Supplemental Material that may be linked to, or accessed through, an article. The authors have granted ASM a non-exclusive, world-wide license to publish the Supplemental Material files. Please contact the corresponding author directly for reuse.
